# Modulation of Intermuscular Beta Coherence in Different Rhythmic Mandibular Behaviors

**DOI:** 10.3389/fnhum.2020.00302

**Published:** 2020-07-30

**Authors:** Evan R. Usler, Xiaomei Wei, Meg Simione, Brian Richburg, Kaila L. Stipancic, Jordan R. Green

**Affiliations:** ^1^Department of Communication Sciences and Disorders, College of Health Sciences, University of Delaware, Newark, DE, United States; ^2^Department of Rehabilitation Medicine, The Third Affiliated Hospital of Sun Yat-sen University, Guangzhou, China; ^3^Department of Pediatrics, Massachusetts General Hospital, Boston, MA, United States; ^4^Speech and Feeding Disorders Laboratory, MGH Institute of Health Professions, Boston, MA, United States

**Keywords:** intermuscular coherence, sensorimotor integration, beta band, mandible, chewing

## Abstract

**Introduction:**

Jaw movement during chewing and speech is facilitated by neural activation patterns for opening and closing movements of the mandible. This study investigated anatomic- and task-dependent differences in intermuscular coherence (IMC) and their association with the parameters of jaw muscle activity using surface electromyography (sEMG).

**Methods:**

We recorded sEMG activation from bilateral and ipsilateral jaw-closing muscle pairs during non-nutritive and nutritive chewing, and during a syllable repetition task. IMC and cross-correlational analyses between bilateral and ipsilateral muscle pairs were performed.

**Results:**

Intermuscular coherence in the beta band was statistically significant between agonist jaw-closing muscle pairs, with beta IMC weaker for rapid syllable repetition compared to chewing tasks. Cross-correlational analysis of muscle co-activation, as well as sEMG burst amplitude, was positively associated with beta IMC strength.

**Discussion:**

Beta IMC was influenced heavily by task-dependent behavioral goals and physiologic demands, which was interpreted as evidence of shared neural drive among jaw-closing muscles.

## Introduction

Learning to speak and chew involves the gradual tuning of the complex muscle group that control mandibular movements (Green et al., 1997; Simione et al., 2018). Although jaw closing is an integral component of both speech and chewing, the coordination of jaw-closing muscles varies significantly across these behaviors to accommodate their divergent behavioral goals and physiologic demands (Moore, 1993; Simione and Green, 2018). During speech, jaw-closing movements transport the lower lip and tongue toward the palate through low-amplitude and relatively tonic muscle activation patterns (Barlow and Rath, 1985; Moore, 1993). During chewing, the jaw-closing muscles are activated in phasic bursts that alternate with those of the jaw-opening muscle. Task differences have not only been observed in the temporal coordination of jaw muscle activation patterns, but also in the intermuscular coherence (IMC) between jaw muscles (Smith and Denny, 1990; Steeve and Price, 2010).

Intermuscular coherence is a correlation of electromyographic activation in the frequency domain between two muscles. The strength of IMC is an indicator of common neural drive to motor neuron pools, which is hypothesized to be generated by shared or synchronized inputs from descending motor pathways (Farmer et al., 1993). In studies of limb muscle coordination, IMC specific to the beta band (∼15–35 Hz) has been reported to be an indicator of coordinated neural drive across functionally linked muscles originating from the motor cortex (Reyes et al., 2017). In a large sample of typical adults (Jaiser et al., 2016), the strength of beta IMC was found to be consistent across adulthood and to be associated with motor performance. Stronger beta IMC may reflect greater synchrony in neural oscillations that are transmitted to motor neuron pools (Flood et al., 2019), resulting in motor unit synchronization driving the coordination of jaw muscles. According to Kerkman et al. (2018), beta IMC distribution patterns across the body indicate functional connectivity across muscles and are strongly shaped by (1) anatomical constraints such as muscle distance and homology, and (2) physical and cognitive demands of the task. Similar anatomical constraints and task-dependent effects on IMC may be evident between jaw muscles during mandibular movement.

Prior works on jaw muscle activation patterns have demonstrated task-dependent effects on IMC. Smith and Denny (1990), for example, found strong IMC between the bilateral masseter muscles of adults during various mandibular behaviors, with IMC between 20 and 60 Hz stronger during chewing than during speech or jaw clenching. Steeve and Price (2010) similarly reported stronger IMC in bilateral masseter and temporalis pairs in an infant and an adult for chewing compared to vocalization, suggesting chewing was facilitated by stronger IMC compared to speech-like behavior. Given that task- and muscle-related differences in the coordinative organization across jaw muscles has also been found in cross-correlational analyses of the degree of muscle co-activation, the strength of IMC during oromotor tasks may be an indicator of common drive to motor neuron pools from “a central command system” involving input from a central pattern generator (Lund, 1991) or reflex pathways (Moore et al., 1988; Moore, 1993; Steeve and Moore, 2009).

Beta IMC has been reported to be modulated by the extent to which a motor task engages afferent feedback, with coherence becoming weaker when afferent sensory feedback is restricted (Fisher et al., 2002). The contribution of afferent feedback to IMC strength suggests a role for sensorimotor integration between afferent sensory information and the efferent neural control of oromotor tasks. In regard to speech, the role of auditory and somatosensory feedback and feedforward mechanisms has been well established (Guenther, 2016). It remains unclear if beta IMC is sensitive to task- and muscle-related differences in the reliance on sensory feedback underlying movement of the jaw. Compared to mandibular control during speech, the coordination of jaw muscles for the manipulation of an object or bolus (such as during chewing) arguably involves a greater reliance on somatosensory feedback (Lund and Kolta, 2006) and thus may be facilitated by strong IMC between synergistic jaw-closing muscles. IMC is also modulated by extramotor factors such as task-dependent cognitive demands. Kristeva-Feige et al. (2002) found beta corticomuscular coherence to weaken when attention is divided and motor precision is decreased during an isometric constant force task. During speech, Stepp et al. (2011) found beta IMC between anterior neck muscles to weaken when participants’ attention was divided (counting backward) compared to a normal speaking condition. Tasks that tax cognitive and motoric resources necessary for mandibular control, such as those characteristic of speech production, may weaken IMC relative to less demanding non-speech tasks such as chewing.

Our understanding of IMC in the beta band as an indicator of common neural input driving functional connectivity between jaw muscles will be strengthened by additional information about the influence of (1) anatomic relations (e.g., bilateral versus ipsilateral muscle pairs), and (2) task-dependent demands in sensorimotor integration and extramotor factors (e.g., chewing versus speech). We recorded muscle activation during non-nutritive (i.e., gum) and nutritive chewing (i.e., food) using sEMG from bilateral and ipsilateral jaw muscle pairs. IMC was also examined during a rhythmic speech-like behavior—a rapid syllable repetition task. Combined, these behaviors elicit a wide range of behavioral demands on agonist jaw muscles that likely result in across-task and within-task differences in beta IMC.

Given the likely influence of sensory input and cognitive demands on beta IMC strength mentioned above, we hypothesized that beta IMC would be stronger for chewing compared to speech-like rapid syllable repetition. We also hypothesized that differences in anatomical function would contribute to task-dependent differences in beta IMC. For example, gum chewing (which was restricted to the right working side) was expected to exhibit a strong beta IMC between right ipsilateral muscles (temporalis and masseter) compared to bilateral pairs. We also hypothesized that beta IMC strength between jaw muscles during these tasks would be correlated with cross-correlational measures of muscle co-activation (peak cross-correlation coefficient and temporal lag) underlying mandibular control. Lastly, as an exploratory analysis without an *a priori* hypothesis, we investigated the potential relationship between beta IMC and sEMG burst amplitude, to determine if this parameter contributed to beta IMC strength.

## Materials and Methods

### Participants

Ten healthy volunteer participants (aged 18–45 years) including seven females and three males were recruited for this study. All participants had a negative history of speech, language, or hearing disorders. All participants also had no history of neurological/musculoskeletal disease or dysfunction that would affect oromotor behavior. All procedures were approved by the Institutional Review Board of Spaulding Rehabilitation Hospital, and all participants gave informed consent. Two additional participants initially participated in the study; however, significant movement artifact during the repetition task resulted in the removal of their data from further analysis.

### Surface Electromyography Recordings

Electromyography recordings were obtained from jaw-closing muscles including right and left masseter and the right and left temporalis. Electrode placement was similar to previous studies (e.g., Green et al., 1997) and determined by palpation of participants’ muscle during jaw clenching. Electrodes were spaced approximately 0.5 cm apart and aligned parallel to muscle fiber orientation. A single ground electrode was placed on the mastoid process. Using the BIOPAC M150 system (BIOPAC Systems, Inc., Goleta, CA, United States), sEMG signals were digitized at a 7792 Hz sampling rate and amplified at a factor of 1000 (gain) with hardware high-pass (10 Hz) and low-pass (5000 Hz) filtering. Simultaneous video and audio signals from the sEMG samples were recorded and reviewed to aid in the removal of artifact and other non-chewing motor behaviors, such as swallowing.

### Tasks

**FIGURE 1:**
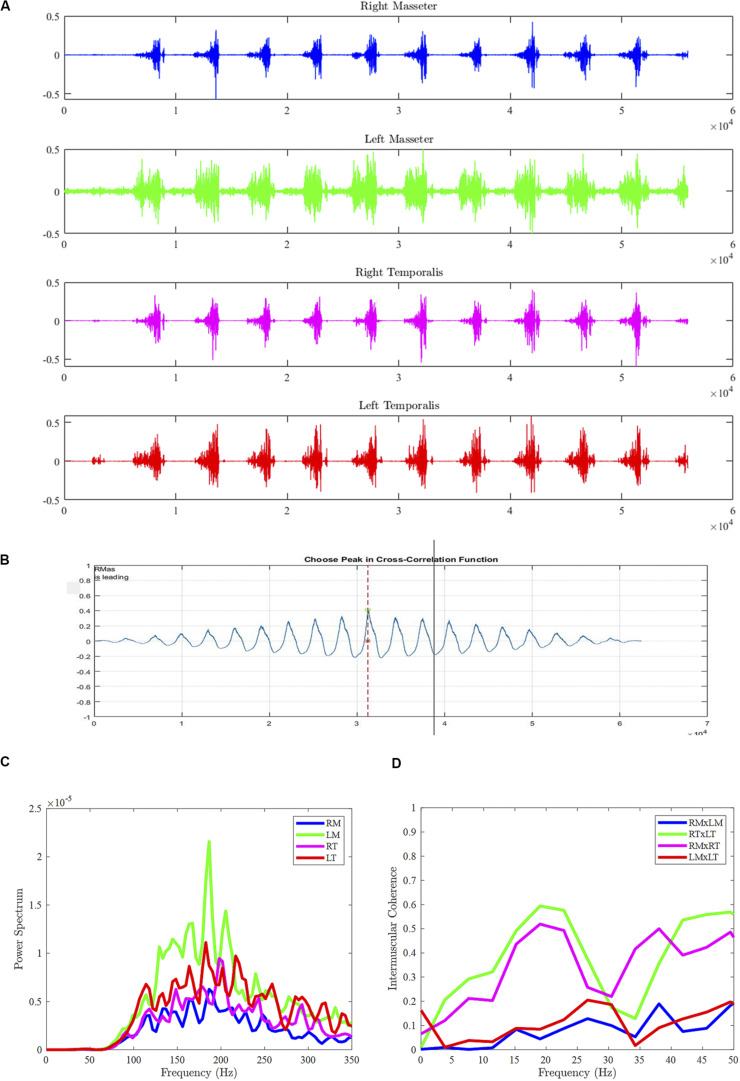
Sample data from Participant #1 during gum chewing (food chewing and syllable repetition tasks not shown), including sEMG bursts across muscles **(A)**, cross-correlogram between right and left masseters **(B)**, power spectral density for each muscle **(C)**, and intermuscular coherence between muscle pairs **(D)**. RM, right masseter; LM, left masseter; RT, right temporalis; LT, left temporalis.

In one experimental session, participants conducted three jaw movement tasks: chewing gum, chewing food, and a rapid syllable repetition task. The ordering of these tasks was consistent across participants to accommodate other experimental conditions beyond the scope of the current study. This acknowledged limitation is discussed further in the Limitations section. Gum chewing was a self-paced and ipsilateral task (chewing only on the right side). A piece of gum was placed on the right molar, and participants were instructed to chew normally. The gum was softened before the task began to ensure consistency during chewing. Food chewing was a self-paced and adaptive bilateral task—three Cheerios (General Mills) were offered twice and participants voluntarily chewed the Cheerios at their own pace until they were ready to swallow. Cheerios were selected as the representative food because it was universally recognized and edible for our participants. Obtaining sEMG parameters for the chewing of Cheerios in typical adults is also important for future studies by our laboratory involving the safe consumption of this food by populations with motor speech and feeding disorders. For the rapid syllable repetition task, participants repeated the syllable/ba/as clearly and quickly as possible on one breath. All the tasks were conducted without any visual or augmented auditory feedback. Each sEMG recording was seven seconds in length, regardless of the task (except for the repetition task for Participant #4 and gum chewing for Participant #3, whose recordings were less than seven seconds). This length of time allowed for a number of chewing cycles (i.e., sEMG bursts) is typical for the breakdown of soft solid foods (e.g., Fontijn-Tekamp et al., 2004). All recordings exhibited at least 10 chewing cycles (see Figure 1A), with the exception of gum chewing by Participants #3 and #8 who produced less than 10 cycles. There was an approximately 3-min interval between each task.

### Data Analysis

#### Intermuscular Coherence Analysis

The continuous sEMG recordings from each task were trimmed according to the reference video and analyzed using MATLAB (MathWorks, Inc., 2009). Audio recordings were used to aid in trimming the sEMG recordings for the rapid syllable repetition task. sEMG activity during vocalization, visible bolus positioning, and swallow movements were excluded from the dataset. The first and last sEMG burst of each recording was also removed from further analysis to remove any potential movement artifact not associated with the chewing cycles. IMC was calculated from continuous and non-rectified sEMG recordings for each task. sEMG recordings were low-pass filtered with an eighth-order Butterworth filter and linearly detrended and amplitude normalized to prevent any slow non-stationarity artifacts to influence calculation of IMC (Boonstra et al., 2009). For each muscle pair, an IMC estimate was calculated using a 2048-point fast Fourier transform and 1948-point Hamming window with 50% overlap. Muscle pairs were yielded, including bilateral agonists: right masseter x left masseter (RMxLM) and right temporalis x left temporalis (RTxLT), and ipsilateral agonists: right masseter x right temporalis (RMxRT) and left masseter x left temporalis (LMxLT). Auto-power and cross-power spectra were calculated using a cross power density (“d”) function in MATLAB. IMC in the beta frequencies (15–35 Hz) was computed as the cross-spectra of the muscle pair normalized by the product of their autospectra (Halliday and Rosenberg, 1999; see Figures 1C,D). Visual inspection of raw sEMG recordings during data collection and analysis was done to ensure signal quality. For example, it was determined that none of the sEMG recordings from our participants exhibited significant noise or sEMG crosstalk (i.e., high degree of IMC across all frequencies).

#### sEMG Parameters and Cross-Correlational Analyses

Cross-correlational analyses—peak cross-correlation and temporal lag to peak cross-correlation coefficient—were also employed as measurements of co-activation between paired jaw muscles (e.g., Green et al., 1997). Participant data were analyzed using a custom MATLAB program (SMASH), which has been described in a previous study (Green et al., 2013). All raw sEMG signals were full-wave rectified, detrended, and low-pass filtered with cutoff of 30 Hz to generate an amplitude envelope for visualization of burst pattern. Pairwise cross-correlations were performed for each muscle pair in a spatiotemporal coupling window in SMASH (see Figure 1B). The peak cross-correlation coefficient was considered an estimate of the strength of activation coupling between the two muscles. Lag to the peak cross-correlation coefficient provided an indication of the temporal synchrony of related activity between the muscle pair. For each participant, burst amplitude of the muscle pairs were also calculated in SMASH as the average root mean square (RMS) across the rectified sEMG waveform. RMS values were normalized using standard *z*-scores for each participant prior to conducting statistical comparisons.

#### Statistical Analysis

Beta IMC, cross-correlational measures, and sEMG burst amplitude were all computed for the four muscle pairs of each participant. The IMC confidence limit (CL) was calculated based on the formula: CL = 1-0.05^1/(L–1)^ by Rosenberg et al. (1989). The IMC confidence limit (CL) was calculated using the formula: CL = 1-0.05^1/(L–1)^ by Rosenberg et al. (1989) that was modified by Terry and Griffin (2008) to account for the use of overlapping segments (L) in calculation of the auto- and cross-power spectra. Because the values of IMC and peak cross-correlation coefficient are both on a scale of 0 to 1, a Fisher’s *z* transformation was used to normalize these data prior to conducting statistical comparisons across participants and across tasks, *n* represents the number of segments, *Coh(f)* represents the coherence of corresponding frequency:

$$z=\sqrt{\left(2\,n\right)}*\text{Tan}\,h^{-1}\,\sqrt{C\,o\,h\,\left(f\right)}$$

Linear mixed models using restricted maximum likelihood fit were applied to determine the fixed effects of “task” and “muscle pair” on the dependent variable “beta IMC” (“lmer” function in R 3.5.2; R Core Team, 2013). “Participant” was entered as the random effect. These analyses were followed by Tukey’s honest significant difference multiple comparisons using the multcomp package (“ghlt” function; Hothorn et al., 2008). In addition, linear mixed model analyses were conducted to determine potential differences in cross-correlational measures (peak coefficient and associated lag) and sEMG burst amplitude. Lastly, Pearson correlation coefficients were computed to determine if beta IMC strength was associated with cross-correlational measures and sEMG parameters. Statistical significance was determined using an alpha level of *p* < 0.05.

## Results

### Intermuscular Coherence

#### Comparisons Between Tasks

**FIGURE 2:**
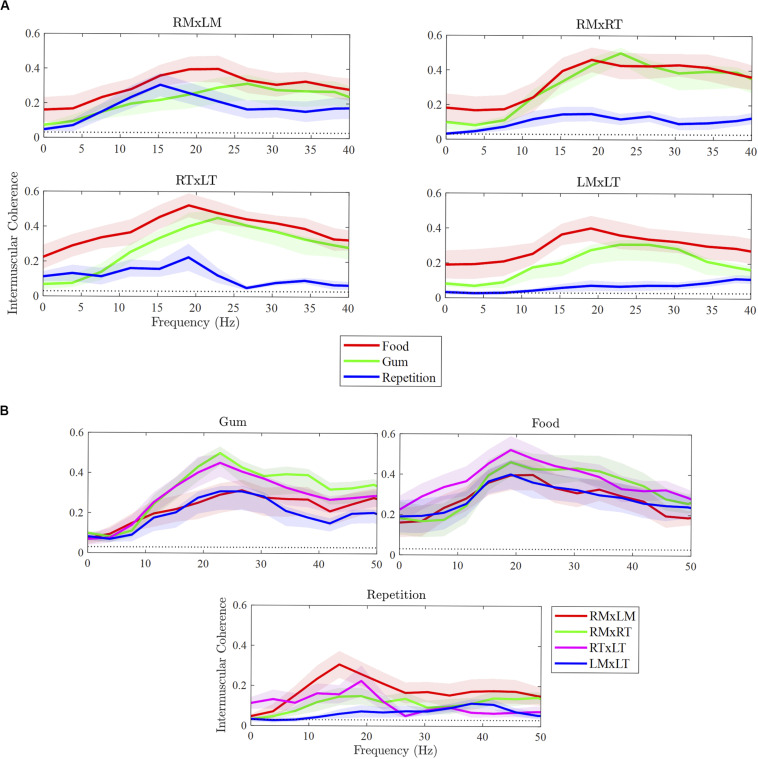
Differences in intermuscular coherence between tasks **(A)** and muscle pairs **(B)**. RMxLM, right masseter x left masseter; RTxLT, right temporalis x left temporalis; RMxRT, right masseter x right temporalis; LMxLT, left masseter x left temporalis.

As illustrated in Figure 2A, the strength of beta IMC differed between tasks for the muscle pairs, with the exception of RMxLM. Overall, beta IMC differed between tasks, *F*(2,108) = 35.35, *p* < 0.001, and pairwise comparisons revealed beta IMC was weaker for rapid syllable repetition compared to chewing food (*p* < 0.001) and chewing gum (*p* < 0.001). The strength of beta IMC did not differ between chewing food and gum (*p* = 0.18). Beta IMC strength was significantly greater during chewing compared to rapid syllable repetition across the muscle pairs, except for RMxLM. Although the chewing of food appeared to exhibit a stronger beta IMC compared to chewing gum across most of the pairs, this difference was not statistically significant.

#### Comparisons Between Muscle Pairs

As illustrated in Figure 2B, beta IMC across muscle pairs was observed in each of the three tasks. Overall, beta IMC differed between muscle pairs for gum chewing, *F*(3,27) = 10.37, *p* < 0.001, and rapid syllable repetition, *F*(3,27) = 3.28, *p* = 0.04, but not for food chewing, *F*(3,27) = 1.77, *p* = 0.18. For gum chewing, pairwise comparisons revealed beta IMC to be stronger for the right ipsilateral muscle pair (RMxRT) compared to left ipsilateral pair (LMxLT; *p* < 0.001) and bilateral masseter pair (RMxLM; *p* < 0.001). The bilateral temporalis pair (RTxLT) was also strong in beta IMC compared to the LMxLT (*p* = 0.003) and RMxLM (*p* = 0.006). For rapid syllable repetition, beta IMC was strong in the RMxLM pair compared to the left ipsilateral (LMxLT) pair (*p* = 0.01), which was relatively low for this task.

### sEMG Parameters

**FIGURE 3:**
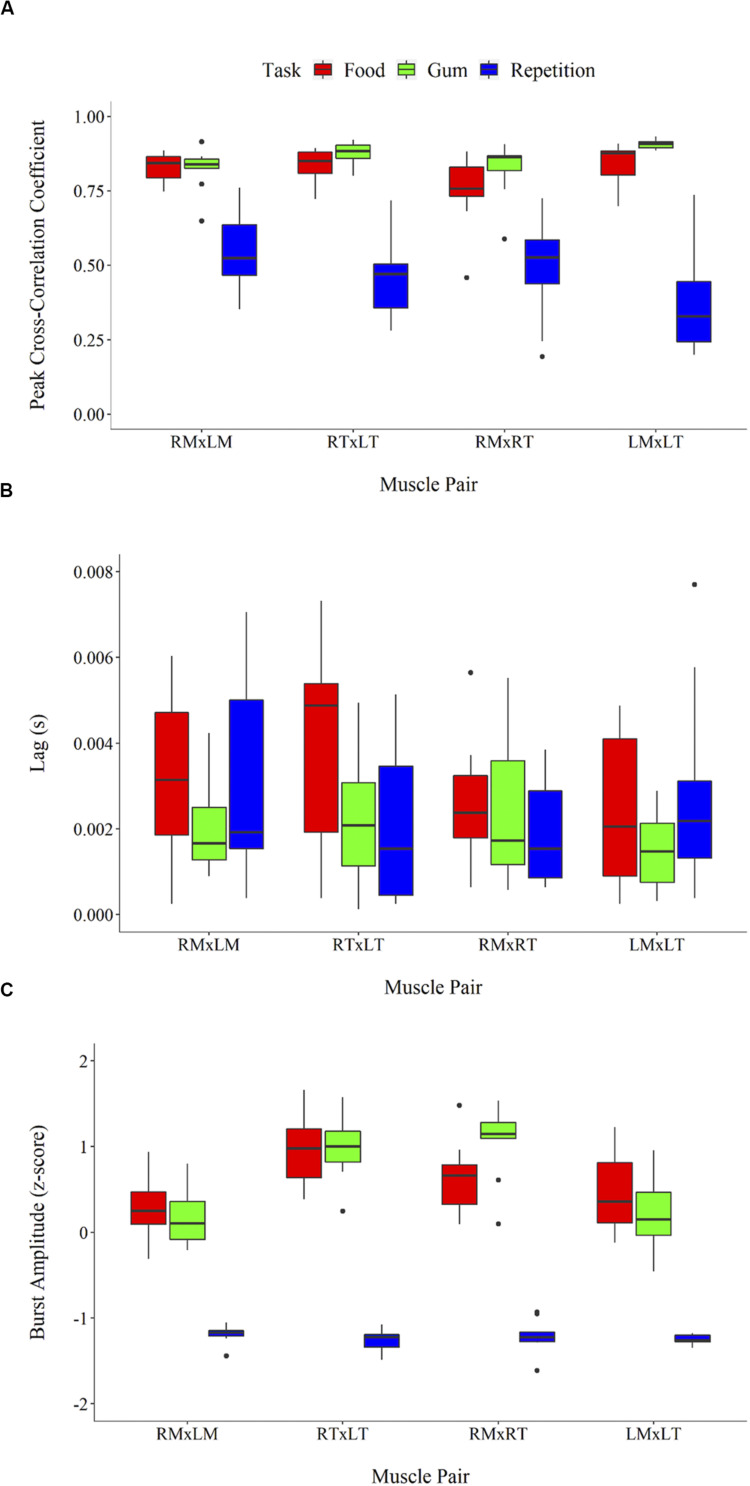
sEMG parameters including peak cross-correlation coefficient **(A)**, associated lag (in seconds) **(B)**, and burst amplitude (*z*-score root mean square) **(C)**. Error bars represent standard error.

Cross-correlational analyses revealed sEMG activation to be tightly coupled across muscle pairs during the chewing tasks (reflected by peak cross-correlation coefficients, *r*s > 0.75), but less so for rapid syllable repetition (*r*s < 0.70). As shown in Figure 3A, peak cross-correlation coefficients differed between tasks, *F*(2,108) = 172.83, *p* < 0.001, and were larger overall for the chewing of gum and food compared to repetition (*p*s < 0.001). Peak cross-correlation coefficients did not differ between the two chewing tasks (*p* = 0.12). Peak cross-correlation coefficients also did not differ between muscle pairs, *F*(3,116) = 0.26, *p* = 0.86. As illustrated in Figure 3B, the lag (or temporal synchrony) of muscle co-activation differed significantly between tasks, *F*(2,111) = 3.33, *p* = 0.86 (*p* = 0.04), but not between muscle pairs, *F*(3,110) = 1.51, *p* = 0.22. Pairwise comparisons between tasks revealed greater asynchrony (i.e., increased lag) for chewing food compared to chewing gum (*p* = 0.03). Lag during repetition did not differ significantly from that during food chewing (*p* = 0.23) and gum chewing (*p* = 0.67). sEMG burst amplitude of the muscle pairs (Figure 3C) differed significantly across tasks, *F*(2,117) = 231.23, *p* < 0.001), but not muscle pairs *F*(3,116) = 1.71, *p* = 0.17). Pairwise comparisons revealed burst amplitude to be lower for rapid syllable repetition compared to the chewing tasks (*p*s < 0.001). Amplitude did not differ between the chewing of food and gum (*p* = 0.99).

**FIGURE 4:**
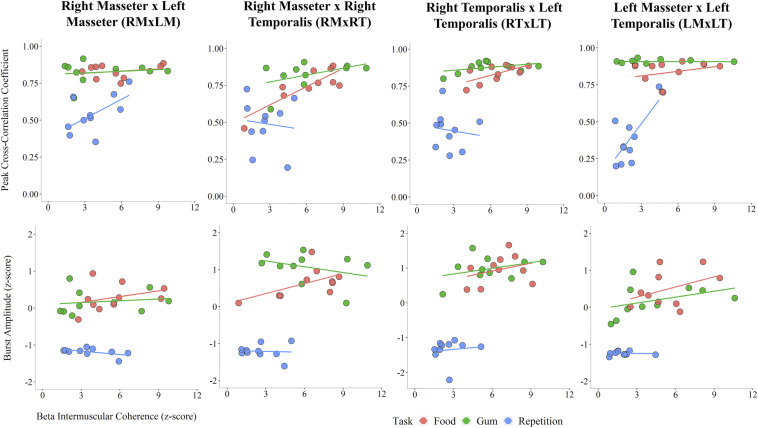
Scatterplots across muscle pairs between beta intermuscular coherence (*z*-score) and peak cross-correlation coefficient **(Top)** and sEMG burst amplitude (*z*-score; **Bottom**).

An exploratory analysis of potential associations between beta IMC and sEMG parameters was performed. Linear regression lines in Figure 4 highlight task differences in the relationship between beta IMC and values of peak cross-correlation coefficient and sEMG burst amplitude. Overall, peak cross-correlation coefficients were positively correlated with beta IMC across the muscle pairs (*r*s > 0.36, *p*s ≤ 0.05). Beta IMC was also positively correlated with RMS amplitude across the muscle pairs (*r*s > 0.29, *p*s ≤ 0.10).

## Discussion

In this study, we recorded sEMG activation from bilateral and ipsilateral jaw-closing muscle pairs to examine differences in beta IMC between non-nutritive (i.e., gum) and nutritive chewing (i.e., food), as well as during rapid syllable repetition. The three tasks elicited differences in beta IMC, cross-correlational measures of muscle co-activation, and sEMG burst amplitude. Across the muscle pairs, beta IMC was moderately positively correlated with the peak cross-correlation coefficient and burst amplitude. These associations between IMC and sEMG activation allowed us to speculate about the functional significance of IMC on mandibular control. Beta IMC in jaw-closing muscles was influenced heavily by task-dependent behavioral goals and physiologic demands. The observed task-dependency is consistent with prior findings on the task-dependency of jaw-muscle activation coupling (Moore, 1993; Steeve and Moore, 2009). Our findings on beta IMC extend this previous work by identifying the task demands that are driving these changes, which include the level of muscle activation, the synergistic and anatomic relation between muscles, and possibly, the engagement levels of afferent feedback and cognition.

### Differences in Jaw-Closing IMC Across Tasks and Muscles Within Tasks

Task- and muscle-dependent differences in coordinative organization were evident in the strength of beta IMC across muscle pairs. The most salient task-related difference observed in this study, consistent with our hypothesis, was the considerably weaker beta IMC across all muscle pairs for rapid syllable repetition compared to the two chewing tasks. During rapid syllable repetition, beta IMC was strongest between the left and right masseters, which are the prime movers of the mandible during speech (Moore, 1993). As expected, during right-lateralized gum chewing, beta IMC was strong between the right temporalis and masseter muscle (the working side) compared to the left. Beta IMC was also particularly strong between the temporalis muscles during gum chewing. This is not surprising because the resistance of gum during chewing required activation of temporalis muscles for additional muscle force for jaw closing (Farella et al., 1999). This finding also corresponds with the high muscle coupling and burst amplitudes observed between the temporalis muscles during chewing gum. Beta IMC was similar in strength between the two chewing tasks and mandibular control during the two chewing tasks was characterized by similar jaw-muscle activation patterns (i.e., strong co-activation and high burst amplitude) compared to rapid syllable repetition. In contrast to gum chewing, food chewing was not restricted to the right working side, and as a result, beta IMC is more similar across all the muscle pairs. The chewing of food, relative to gum, was also reduced in muscle co-activation and synchrony. The more vertical and rhythmic cycles that characterized gum chewing (Simione and Green, 2018) was likely aided by a masticatory central pattern generator in the brainstem that may be relatively isolated from the cortical motor drive associated with beta IMC.

The weaker beta IMC during rapid syllable repetition is congruent with previous findings of stronger IMC between masseter muscles during chewing compared to speech-like behavior (Smith and Denny, 1990; Steeve and Price, 2010). The co-activation of muscles (revealed by the peak cross-correlation coefficient) for chewing could be strong even with a relatively low beta IMC. Although speculative, the strong beta IMC characterizing the chewing tasks may be an indicator that jaw movement during chewing may be, at least in part, controlled by a brainstem central pattern generator (Smith and Denny, 1990). Unlike the chewing tasks, muscle activation during rapid syllable repetition was characterized by relatively weak muscle co-activation and reduced burst amplitude, all of which are characteristic of muscle activation during speech (Moore, 1993). Also characteristic of speech is the primary role of the bilateral masseters, evidenced by the strong beta IMC between these muscles during the repetition task compared to the other muscle pairs.

The moderate correlations observed between beta IMC and the parameters of sEMG co-activation and amplitude across the tasks provides evidence that task-dependent differences in sEMG co-activation and amplitude may be associated with beta IMC. More specifically, the sEMG activation patterns characteristic of chewing likely contributed to the relatively strong beta IMC that was observed, and vice versa for the relatively weak beta IMC during rapid syllable repetition. As indicated by higher sEMG amplitudes, chewing required more force generation than speech (Barlow and Rath, 1985). Given that greater force generation is associated with stronger corticomuscular coherence in the beta frequency (Witte et al., 2007), the relatively greater force generation characteristic of chewing may have contributed to the task-dependent differences in beta IMC.

In addition, the weaker beta IMC observed during rapid syllable repetition compared to the chewing tasks may also have also been influenced by differences in cognitive demands (Simione and Green, 2018). The current findings raise the possibility that beta IMC may be stronger for tasks that are highly practiced, such as chewing, than for are motorically and cognitively demanding such as the rapid syllable repetition task, a task that is designed to elicit maximum performance. In sum, differences in neuromuscular activation, task dynamics, anatomical relations, and cognitive demands all likely contribute to differences in beta IMC, revealing varieties in the neural control of different mandibular behaviors.

### Limitations and Future Research Considerations

The current study was limited by the consistent ordering of the tasks, which was done in order to accommodate other experimental conditions beyond the scope of the current study. However, the evidence provided of task-related effects in beta IMC between muscle pairs, which were not likely affected by any potential order effects, is suggestive that the observed differences in beta IMC were likely due to the task itself and not the order of their production. Still, future studies should randomize these tasks to prevent any order effects. The limited number of trials in the current study was necessary because (1) chewing of a simple food substance, such as Cheerios, prevented a large amount of chewing cycles, and (2) this was a preliminary study to determine the appropriateness of such a paradigm with populations with bulbar motor dysfunction who are not able to provide large numbers of chewing cycles without fatigue. Given that coherence values have been shown to either increase or remain stable with the use of increasing segments for coherence calculation (van Asseldonk et al., 2014), future studies consisting of jaw movement tasks with a feasibly greater number of trials are necessary to determine the reliability of IMC as a marker of neural control of mandibular behavior.

## Data Availability Statement

The datasets for this article are not publicly available because prior approval to share the data was not obtained from the research participants. Requests to access the datasets should be directed to JG, jgreen2@mghihp.edu.

## Ethics Statement

The studies involving human participants were reviewed and approved by the MGH Institutional Review Board. The participants provided their written informed consent to participate in this study.

## Author Contributions

Data interpretation and subsequent drafting of this article were conducted by EU, XW, and JG. Furthermore, data were collected by MS and XW. JG and MS provided the experimental conception and design of the study. All authors contributed to the article and approved the submitted version.

## Conflict of Interest

The authors declare that the research was conducted in the absence of any commercial or financial relationships that could be construed as a potential conflict of interest.

## Abbreviations

sEMG: surface electromyography
IMC: intermuscular coherence
LMxLT: left masseter x left temporalis pair
RMxLM: right masseter x left masseter pair
RMxRT: right masseter x right temporalis pair
RTxLT: right temporalis x left temporalis pair
RMS: root mean square.
